# Effects of virtual reality interventions on motor skills in children and adolescents with intellectual disabilities: a systematic review and meta-analysis

**DOI:** 10.3389/fpsyt.2026.1919592

**Published:** 2026-08-25

**Authors:** Ling Qin, Zhe Zhang, Bing Xu

**Affiliations:** 1 Faculty of Physical Education and Health Science, Chongqing Normal University, Chongqing, China; 2 Faculty of Sports and Exercise Science, Universiti Malaya, Kuala Lumpur, Malaysia; 3 Faculty of Physical Education, Jiangxi Institute of Technology, Nanchang, China

**Keywords:** intellectual disability, motor skills, virtual reality, youth, children

## Abstract

**Background:**

Virtual reality (VR) technology is increasingly applied in rehabilitation and adapted physical activity programmes for children and adolescents with intellectual disabilities (ID). However, evidence on the efficacy of VR-based interventions in improving motor skills is limited and inconclusive.

**Objective:**

This systematic review and meta-analysis aimed to evaluate the effects of VR-based interventions on motor skills in children and adolescents with ID and summarise the methodological quality of the available evidence.

**Methods:**

Seven electronic databases were searched for English-language VR intervention studies involving children and adolescents with ID or related conditions affecting intellectual function. Study selection, data extraction, and risk-of-bias assessment were conducted systematically. Methodological quality was assessed using the Cochrane Risk of Bias 2 tool. Where sufficient data were available, a meta-analysis was performed to estimate the pooled effect sizes, and subgroup analyses were conducted to compare the immersive and non-immersive VR interventions.

**Results:**

Ten studies involving 414 participants were included. VR interventions had significant positive effects on gross motor skills, locomotor skills, object-control skills, balance, agility and strength. Subgroup analyses indicated favourable effects for both immersive and non-immersive VR; significant subgroup differences were observed in gross motor skills.

**Conclusion:**

VR-based interventions may improve motor-related outcomes in children and adolescents with ID, although effect robustness varied across domains and the apparent advantage of immersive VR remains exploratory. Adequately powered, rigorously designed randomised controlled trials with standardised outcomes are needed to confirm these findings.

**Systematic review registration:**

https://www.crd.york.ac.uk/PROSPERO/view/CRD420251088479, identifier CRD420251088479.

## 1 Introduction

Intellectual disability (ID) is a neurodevelopmental disorder with an onset before age 22, characterised by significant limitations in intellectual functioning and adaptive behaviour across conceptual, social, and practical domains (1). These limitations may persist across the lifespan and are commonly associated with difficulties in academic attainment, communication and social participation, independent daily living, and occupational functioning, typically necessitating ongoing support of varying intensities. Approximately 3.2% of children and adolescents worldwide meet the diagnostic criteria for ID (2).

Physical activity (PA) is essential for the physical, psychological, emotional, and cognitive development of children and adolescents, regardless of their ID status (3). It promotes overall health and well-being (4). According to the WHO guidelines (5), children and adolescents with disabilities should have an average of at least 60 minutes per day of moderate-to-vigorous PA across the week, consistent with recommendations for their typically developing peers.

Despite its well-documented benefits, children and adolescents with ID engage in insufficient PA and encounter multiple barriers to participation (6). Consequently, they often exhibit lower physical fitness across several domains, including cardiorespiratory endurance, muscular strength and endurance, speed, balance, and agility, than peers without ID, alongside elevated rates of overweight and obesity (7). These adverse outcomes can be modified by increased PA engagement (8, 9). Accordingly, elucidating the determinants of PA behaviour is essential for long-term health promotion in this population.

Motor skills competence refers to the mastery of fundamental movement patterns and goal-directed actions involving large muscle groups or the whole body (10). Proficiency in motor skills enables the acquisition of more complex movement patterns and supports participation in organised sports across the lifespan (11, 12). The relationship between PA and motor skills is reciprocal and develops across childhood and adolescence. Greater PA affords more practice opportunities and skill refinement, whereas higher competence enhances success and enjoyment, further promoting PA (13). Stronger early motor skills predict higher PA levels later in development (14, 15). Furthermore, greater competence in motor skills is associated with better cardiorespiratory fitness and healthier weight status, and is positively related to language development, executive function, and academic achievement (16–18).

Virtual reality (VR) is a computer-generated simulation that employs specialised hardware and software to construct immersive representations of real or imagined environments (19). The rapid growth of internet-based platforms, assistive devices, mobile applications, and wearable sensors has significantly expanded the range of technology-mediated interventions. These digital tools are now applied across numerous health contexts (20–22), including strategies designed to improve motor skills in children and adolescents with ID (23).

VR technology is classified into immersive and non-immersive modalities. Immersive VR blocks the user’s perception of the physical environment and creates a fully computer-generated experience, whereas non-immersive VR delivers virtual content through standard screens and is often implemented as a serious or active video game (24). Both immersive and non-immersive VR technology are feasible for motor skill interventions among children and adolescents with ID (25–27). Moreover, VR offers children and adolescents with ID a secure and highly controlled environment (28). Furthermore, it supports tailoring interventions to individual needs while progressively introducing task complexity (29).

Prior studies suggest that VR technologies enhance the motor skills of children and adolescents with ID. However, several important knowledge gaps remain. First, to the best of our knowledge, no systematic review or meta-analysis has yet synthesised evidence on the effects of VR interventions on motor-skill outcomes in this population. Second, although VR has been considered a practical approach for motor-skill interventions in children and adolescents with ID (26, 27, 30), evidence regarding whether immersive and non-immersive VR produce different effects remains limited. Third, the magnitude of VR effects on specific motor-skill domains remains unclear. Existing studies have assessed diverse outcomes, such as gross motor, locomotor, and object-control skills, agility, balance, and strength-related performance (30–32), but domain-specific effect sizes have not been systematically quantified.

This study aims to systematically review and meta-analyse the effects of VR interventions on motor-skill outcomes in children and adolescents with ID and has three objectives. First, to synthesise the available evidence on the overall effectiveness of VR interventions for improving motor skills in children and adolescents with ID. Second, to compare the effects of immersive and non-immersive VR interventions on motor-skill outcomes in this population. Third, to estimate the effects of VR interventions on domain-specific motor skills.

## 2 Materials and methods

This systematic review and meta-analysis was conducted in accordance with the PRISMA guidelines (33). The protocol was registered with the International Prospective Register of Systematic Reviews (PROSPERO), registration number CRD420251088479.

### 2.1 Search strategy

The following electronic bibliographic databases were searched: CINAHL, MEDLINE, PsycINFO, PubMed, Scopus, SPORTDiscus, and Web of Science. The final searches were completed on 5 April 2026, using search terms related to the participants, intervention, and outcome. Only human studies published in English and peer-reviewed journals were included. The same search strategy was used for all databases (**Table 1**). Finally, the references of eligible studies and relevant articles were searched for backwards and forward citations.

**Table 1:** Search strategy.

Search components	Search terms
Participant	Intellectual Disability OR Intellectual Disabilities OR Intellectual Development Disorder OR Development Disorder OR Intellectual Disorder OR Intellectual Development Disorders OR Mental Retardation OR Idiocy OR Psychosocial Mental Retardation OR Mental Deficiencies OR Mental Deficiency OR mental handicap OR Cognitive Disability OR Cognitive Impairment OR Cognitive Deficit OR Cognitive Disorder OR Global Developmental Delay OR Developmental Delay OR Cri-du-Chat Syndrome OR De Lange Syndrome OR Down Syndrome OR Prader-Willi Syndreome OR Rubinstein-Taybi Syndrome OR Trisomy 13 Syndrome OR WAGR Syndrome OR Williams Syndrome OR X-Linked Intellectual Disability OR Adrenoleukodystrophy OR Coffin-Lowry Syndrome OR Fragile X Syndrome OR Glycogen Storage Disease Type IIb OR Lesch-Nyhan Syndrome OR Menkes Kinky Hair Syndrome OR Mucopolysaccharidosis II OR Pyruvate Dehydrogenase Complex Deficiency Disease OR Rett Syndrome
Intervention	AND
	Virtual Reality OR Educational Virtual Realities OR Educational Virtual Reality OR Instructional Virtual Realities OR Instructional Virtual Reality OR immersive OR non-immersive OR Virtual Game OR Exergame OR Active Game OR Active Video Game OR Interactive Video game OR Wii OR Xbox OR Kinect OR Play Station OR Android Virtual Device OR Nintendo OR Switch
Outcome	AND
	Motor Skill OR Gross Motor Skill OR Fine Motor Skill OR Object Control OR Coordination OR Locomotor OR Balance OR Agility OR Motor Proficiency OR Motor Competency OR Motor Ability OR Motor Development OR Movement Skill OR Motor Performance OR Motor Fitness OR Fundamental Movement OR Basic Movement OR Motor Coordination OR Motor Control OR Manual Dexterity OR Motor Learning

### 2.2 Inclusion and exclusion criteria

This study included studies that met all of the following criteria: (1) employed a randomized controlled trial (RCT) or a randomized controlled trial/quasi-experimental design with a comparator group to assess the effectiveness of any VR intervention; pilot/feasibility studies were eligible if they adopted an RCT or controlled quasi-experimental design; (2) involved children or adolescents aged 3–18 years; (3) included participants diagnosed with ID, or with other developmental disabilities/health conditions associated with impaired intellectual function, and (4) reported at least one motor-skill outcome (e.g., gross motor skills or object motor skills). Studies were excluded if they (1) lacked a control/comparator group (e.g. single-arm pre–post studies); (2) did not report motor skill outcomes; or (3) were observational studies (cross-sectional, cohort, or case-control), qualitative studies, case reports/series, protocols, reviews, conference abstracts, dissertations/theses, or editorials, or were not published in English.

### 2.3 Study selection process

This study followed a two-stage approach: an initial screening of titles and abstracts, followed by a full-text review of potentially eligible studies. This process was conducted independently by the first and second authors (LQ and ZZ) to minimise bias and ensure reliability of the study selection. In the first stage, titles and abstracts of all records identified through the search strategy were screened against predefined eligibility criteria using a standardised screening tool. Records that did not meet the eligibility criteria were excluded at this stage. If insufficient information in the title and abstract prevented the determination of eligibility, the record was retained for full-text review. Any disagreements between the reviewers were resolved through discussion or, if necessary, consultation with a third reviewer (BX). In the second stage, the full-text articles of all potentially eligible records were retrieved and reviewed. The reviewers independently assessed each full-text article against eligibility criteria using a standardised data extraction form. Any disagreements were resolved through discussion or, if necessary, consultation with a third reviewer (BX). The reasons for excluding studies during the full-text review were recorded. Studies were excluded if they did not meet the eligibility criteria related to the population, intervention, comparator, outcomes, or study design, or if the full text was not available or the article was a duplicate publication.

### 2.4 Data extraction

The following data were extracted from each study by the first reviewer (LQ): first author, year of publication, country, sample size, age, type of disease, intervention characteristics (duration, frequency/week, and period), VR intervention, type of VR, treatment of the control group, assessment, outcome indicators, and effects. Missing data were addressed by contacting the researchers to obtain unreported data or additional details.

### 2.5 Risk of bias

The risk of bias of the included randomised controlled trials was assessed using the Cochrane Risk of Bias 2 tool, which evaluates bias arising from the randomisation process, deviations from intended interventions, missing outcome data, outcome measurement, and selective reporting. Two authors (XB and ZZ) independently performed the assessment. Any disagreements were resolved through discussion with the corresponding author (LQ) to reach a consensus.

### 2.6 Data analysis

Data were analysed using Review Manager 5.4 and Stata 17.0. For continuous outcomes, pooled effect sizes were expressed as standardised mean differences (SMDs) with 95% confidence intervals (CIs). Given the anticipated clinical and methodological diversity in participant characteristics, VR modalities, intervention protocols, and outcome measures, random-effects models were used for all meta-analyses (34). Statistical heterogeneity was assessed using Cochran’s Q test and the I² statistic. Leave-one-out sensitivity analyses were conducted to evaluate the robustness of the pooled estimates and identify potentially influential studies. Publication bias was assessed through visual inspection of funnel plots and Egger’s regression test when at least 10 studies were available (35).

## 3 Results

### 3.1 Literature search results

**Figure 1:**
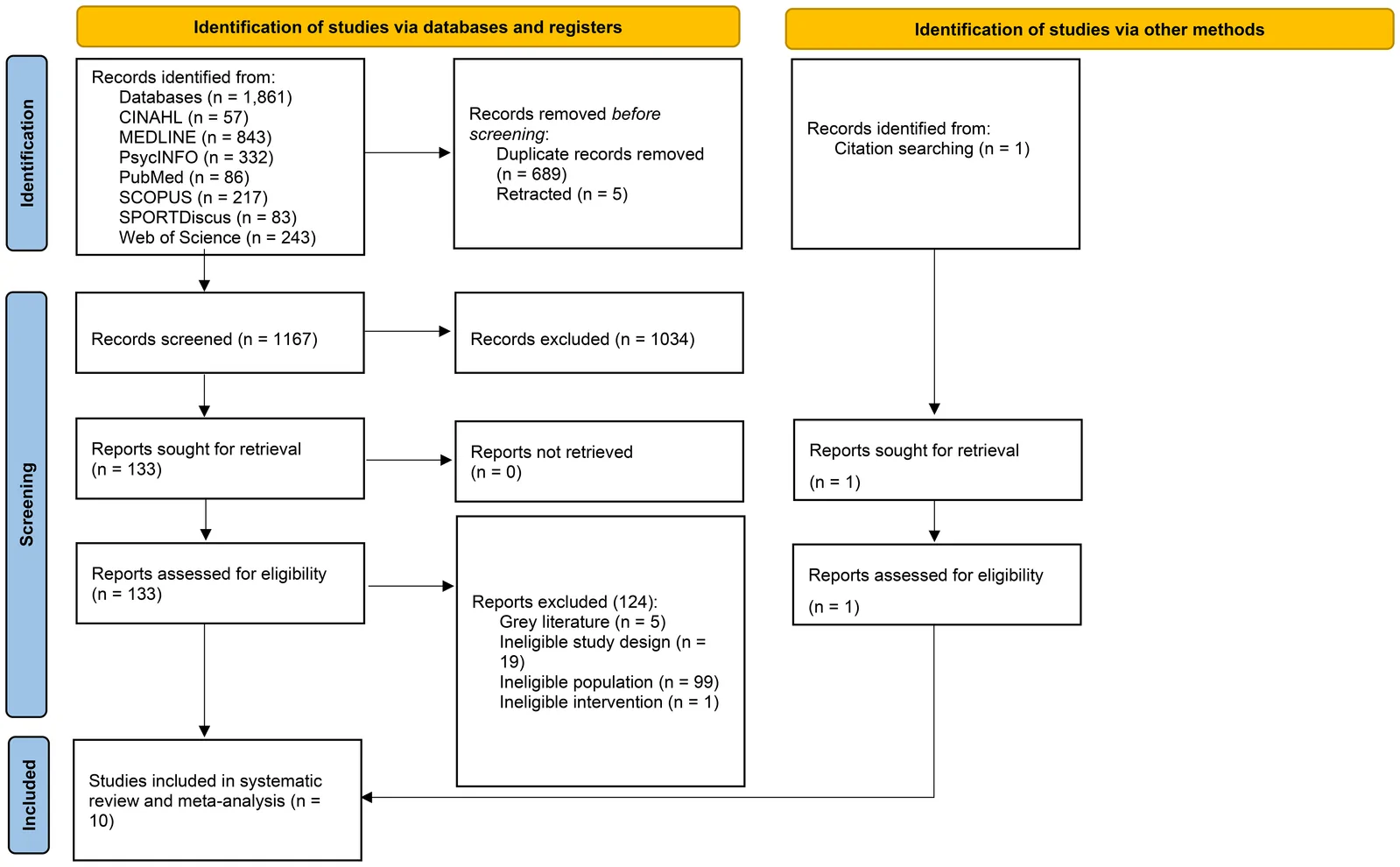
Literature selection process for systematic review.

Seven databases were searched: CINAHL (n = 57), MEDLINE (n = 843), PsycINFO (n = 332), PubMed (n = 86), Scopus (n = 217), SPORTDiscus (n = 83), and Web of Science (n = 243). After removing duplicates and retracted articles, 1, 167 articles remained. After title and abstract screening, 134 articles were retained for full-text review. After full-text assessment, 10 studies were included in the systematic review (26, 27, 30–32, 36–40), of which 10 were further included in the meta-analysis (**Figure 1**).

### 3.2 Basic characteristics of included studies

Ten studies involving 414 participants were included in the meta-analysis. Published between 2011 and 2023, these studies were conducted in Chile, Brazil, Chinese Taiwan, South Korea, France, Spain, and China, and primarily enrolled children and adolescents with Down syndrome and ID. Notably, one study conducted in South Korea enrolled a mixed sample of participants with ID or autism spectrum disorder (30), and another included participants with ID, autism spectrum disorder, or Down syndrome (26). The sample sizes ranged from 12 to 100 participants, and participants’ ages ranged from approximately 5 to 18 years. Most interventions used commercially available non-immersive systems from the Nintendo Wii and Xbox Kinect families. Two studies employed immersive VR exergame systems, using either a projection-based information and communications technology setup or head-mounted displays. In the control groups, participants engaged in usual daily activities, physical education, adapted/special education, or no treatment. Across the included studies, intervention sessions lasting 20–60 min were delivered 2–4 times per week for 4–24 weeks. Outcome assessments were commonly performed using standardised motor-related measures, including TGMD, BOT, KTK, PBS, and functional motor tests, with outcomes mainly focusing on gross motor skills, locomotor and object-control skills, balance, agility, and strength. The basic characteristics of the included studies are presented in **Table 2**.

**Table 2:** Basic Characteristics of the included studies.

Study	Country	Sample size (E/C)	Age (E/C)	Type of disease	Intervention characteristics						Assessment	Outcome indicators	Effect
					Duration (min)	Frequency/week	Period (week)	VR intervention	Type of VR	Treatment of the control group			
Álvarez et al., 2018	Chile	9/7	E:8.30 ± 2.06 C:8.43 ± 1.62	Down syndrome	20	2	5	Nintendo Wii with the Wii Balance Board	Non-immersive	Normal daily activities	TGMD-2	Gross motor skill; Locomotor; Object Control.	Post > Pre
Gouveia Reis et al., 2017	Brazil	7/5	E:9 ± 2.5 C:8 ± 2.5	Down syndrome	20	4	4	Xbox 360 Kinect	Non-immersive	Usual activities	KTK; PBS; Sit-ups	Balance; Strength	Post > Pre
Hsu, 2016	Chinese Taiwan	8/8	E:17.5 ± 0.7 C:17.4 ± 0.5	Intellectual disability (moderate)	40	2	8	Wii Fit balance game	Non-immersive	Physical education	A force plate; Time up and Go; Sit-ups.	Balance; Strength.	Post > Pre
Kwon, 2022	South Korea	27/25	E:10, 15 ± 1.51 C:10.24 ± 1.48	Intellectual disability; Autism spectrum disorder.	No report	No report	12	Projection-based information and communications technology exergame system (multi-sensor tracking)	Immersive	No Treatment	TGMD-3	Gross motor skill; Locomotor; Object Control.	Post > Pre
Lee & Jin, 2023	South Korea	12/11	E:9.9 ± 1.4 C:9.8 ± 1.8	Intellectual disability; Autism spectrum disorder; Down syndrome.	40	2	12	Head-Mounted Display and VR Training System	Immersive	No Treatment	TGMD-3	Gross motor skill; Locomotor; Object Control.	Post > Pre
Lin & Wuang, 2012	Chinese Taiwan	46/46	E:15.6 ± 3.6 C:14.9 ± 3.9	Down syndrome	35	3	6	Nintendo Wii	Non-immersive	No treatment	BOT-2	Strength; Agility.	Post > Pre
Regaieg et al., 2021	France	12/12	E:9.42 ± 1.65 C:8.42 ± 1.08	Intellectual disability (moderate)	60	2	10	Xbox 360 Kinect	Non-immersive	Adapted physical education	TGMD-2	Gross motor skill; Locomotor; Object Control.	Post > Pre
Suarez-Villadat et al., 2023	Spain	24/25	14.19 ± 2.06	Down syndrome	60	3	20	Nintendo Wii	Non-immersive	Special education	Standing long jump; Timed Up and Go Sit-ups.	Balance; Strength.	Post > Pre
Wang et al., 2022	China	15/15	E:13 ± 0.8 C:14 ± 2.2	Intellectual disability (moderate)	40	3	8	Xbox Kinect	Non-immersive	Physical education	Time Up and Go PBS	Balance	Post < Pre
Wuang et al., 2011	Chinese Taiwan	50/50	7-12	Down syndrome	60	2	24	Nintendo Wii	Non-immersive	No treatment	BOT-2	Strength; Agility.	Post > Pre

E, Experimental Group; C, Control group;

TGMD, Test of Gross Development; KTK, Körperkoordinatonstest Für Kinder body assessment test; PBS, Pediatric Balance Scale; BOT, Bruininks–Oseretsky Test of Motor Proficiency.

### 3.3 Quality assessment

**Figure 2:**
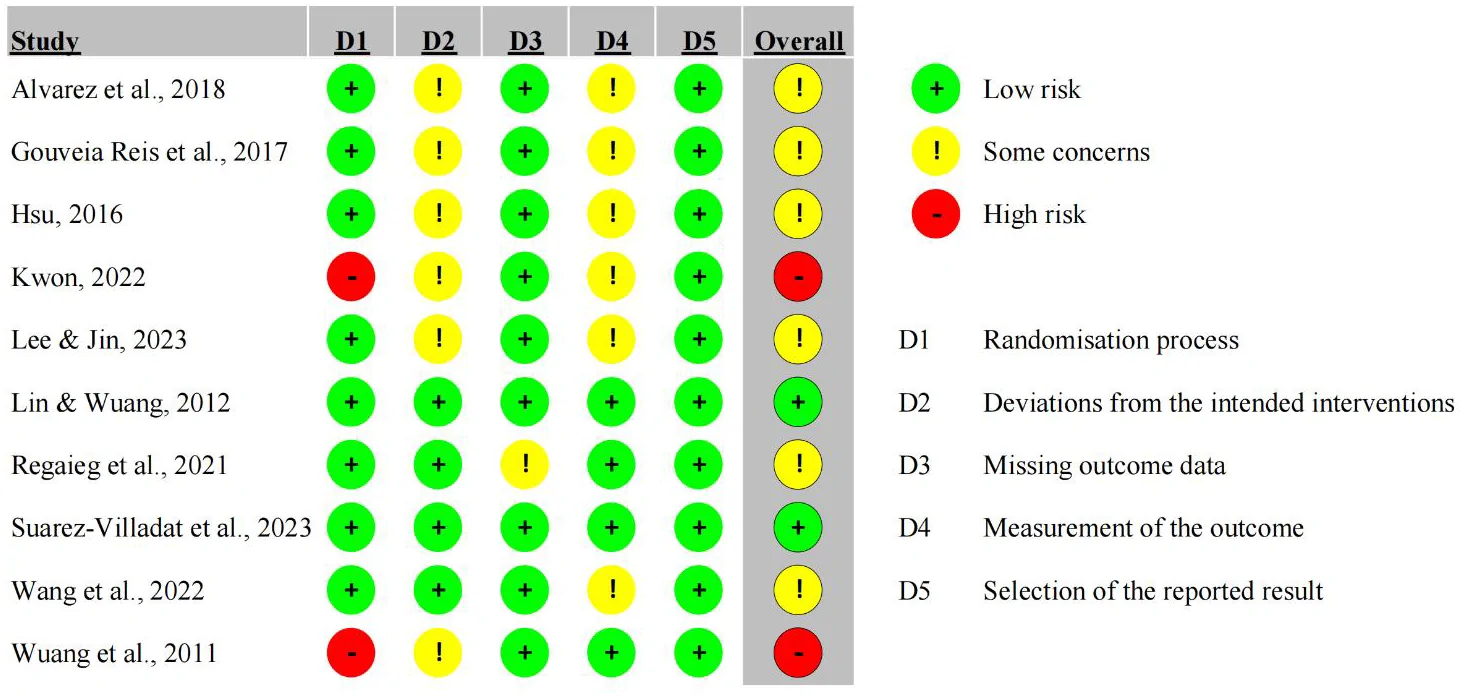
Risk of bias traffic-light plot.

The detailed risk-of-bias assessments are presented in **Figure 2**. Two studies were assessed as low risk (31, 32), six studies showed some concerns (26, 27, 36–39), and two studies (30, 40) showed high risk of bias. Regarding randomisation bias, most studies clearly reported sequence generation; one study used a no-treatment control group that was not randomly allocated (40), and another did not clearly report whether group allocation was randomised (30). Regarding bias from intervention deviations, four studies were judged as low risk as the interventions followed protocol under supervised conditions with no significant departures. The remaining six studies showed some concerns—mainly owing to open-label interventions, unmatched comparator conditions in intensity or attention, or insufficiently controlled outside physical activity/home practice (26, 27, 30, 36–38)—which may have introduced expectancy effects or behavioural compensation. However, no study reported clear evidence of major contamination between groups. Regarding bias related to missing outcome data, one study was rated as having some concerns. Regaieg et al. (39) reported that 6 of 30 selected children did not complete the protocol; however, the timing of these losses relative to randomisation was not clearly described. Nine studies had complete or near-complete follow-up, with no systematic missing-data patterns for outcomes or groups, thereby reducing the risk. Four studies showed low risk of bias in outcome measurements as these assessments used blinded assessors and/or relied on standardised, relatively objective procedures. The remaining six studies were rated as having some concerns (26, 27, 30, 36–38), mainly because blinding of outcome assessors was not clearly reported for process-oriented or performance-based measures. No study was judged as high risk in this domain. All studies reported outcomes according to predefined protocols and time points—with no selective reporting bias—even for outcomes with no significant differences.

### 3.4 Meta-analysis results

Meta-analyses were conducted for the following five motor-skill domains: gross motor skill, locomotor skill, object control skill, balance, and agility. Strength outcomes was analysed separately as secondary outcomes of physical fitness. Ten studies provided sufficient data for the quantitative analyses.

**Figure 3:**
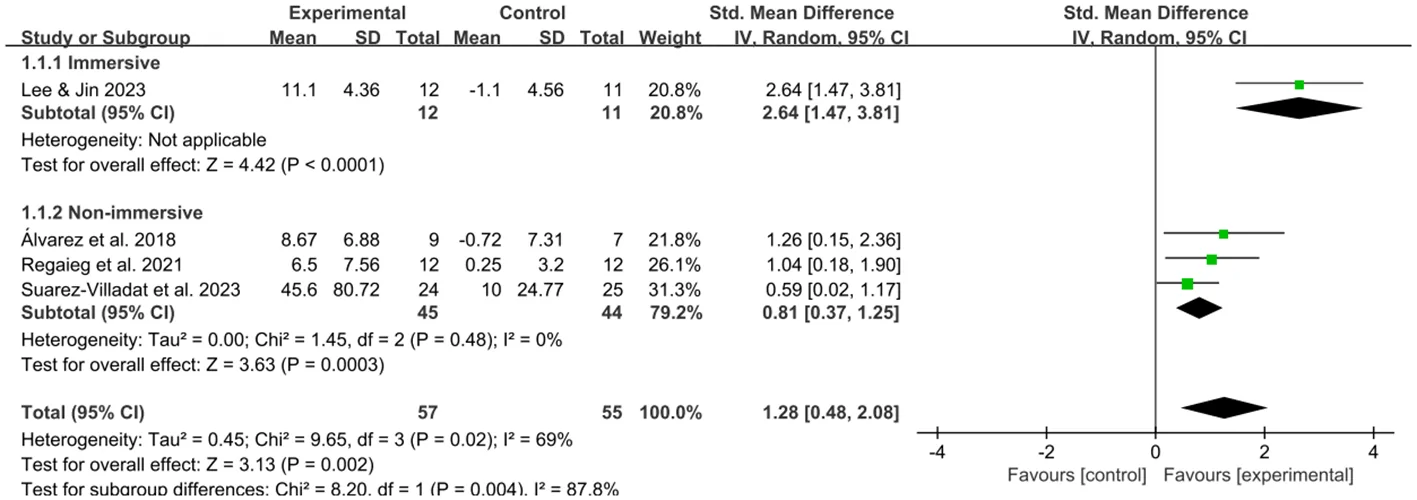
Forest plot of the effects of virtual reality on gross motor skill.

The meta-analysis of gross motor-skill outcomes included four studies (**Figure 3**), with 57 participants in the VR groups and 55 in the control groups. The overall pooled effect indicated a statistically significant positive effect of VR interventions on gross motor skills (SMD = 1.28, 95% CI [0.48, 2.08], Z = 3.13, *p* = 0.002), with substantial heterogeneity across studies (I² = 69%, *p* = 0.02). Only one study, involving 12 participants in the intervention group and 11 in the control group, evaluated immersive VR. This study reported a large and statistically significant effect in favour of immersive VR (SMD = 2.64, 95% CI [1.47, 3.81], Z = 4.42, *p* < 0.0001). In contrast, the non-immersive VR subgroup included three studies with 45 intervention participants and 44 control participants. There was a statistically significant positive effect (SMD = 0.81, 95% CI [0.37, 1.25], Z = 3.63, p = 0.0003) with no observed heterogeneity (I² = 0%, p = 0.48). The test for subgroup differences was statistically significant (χ² = 8.20, *df* = 1, *p* = 0.004), suggesting a larger effect estimate for immersive than for non-immersive VR.

**Figure 4:**
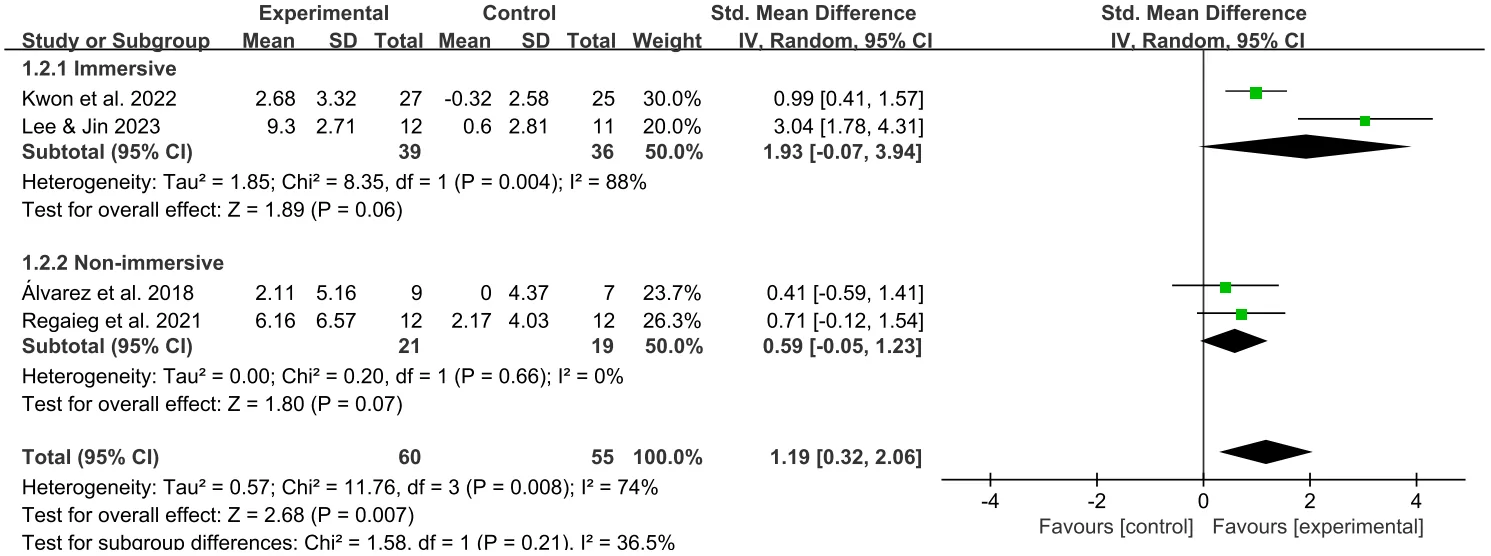
Forest plot of the effects of virtual reality on locomotor skill.

The meta-analysis of locomotor-skill outcomes included four studies (**Figure 4**), with 60 participants in the VR groups and 55 in the control groups. The overall pooled effect indicated a statistically significant positive effect of VR interventions on locomotor skills (SMD = 1.19, 95% CI [0.32, 2.06], Z = 2.68, *p* = 0.007), with substantial heterogeneity across studies (I² = 74%, *p* = 0.008). In the immersive VR subgroup, three studies involving 39 intervention participants and 36 control participants showed no significant positive effect (SMD = 1.93, 95% CI [−0.07, 3.94], Z = 1.89, *p* = 0.06), with high heterogeneity (I² = 88%, *p* = 0.004). The non-immersive VR subgroup also included two studies, comprising 21 participants in the intervention groups and 19 in the control groups. The pooled effect similarly favoured non-immersive VR but was not statistically significant (SMD = 0.59, 95% CI [−0.05, 1.23], Z = 1.80, *p* = 0.07), with no observed heterogeneity (I² = 0%, *p* = 0.66). The test for subgroup differences was not statistically significant (χ² = 1.58, *df* = 1, *p* = 0.21; I² = 36.5%), indicating no significant difference between immersive and non-immersive VR interventions.

**Figure 5:**
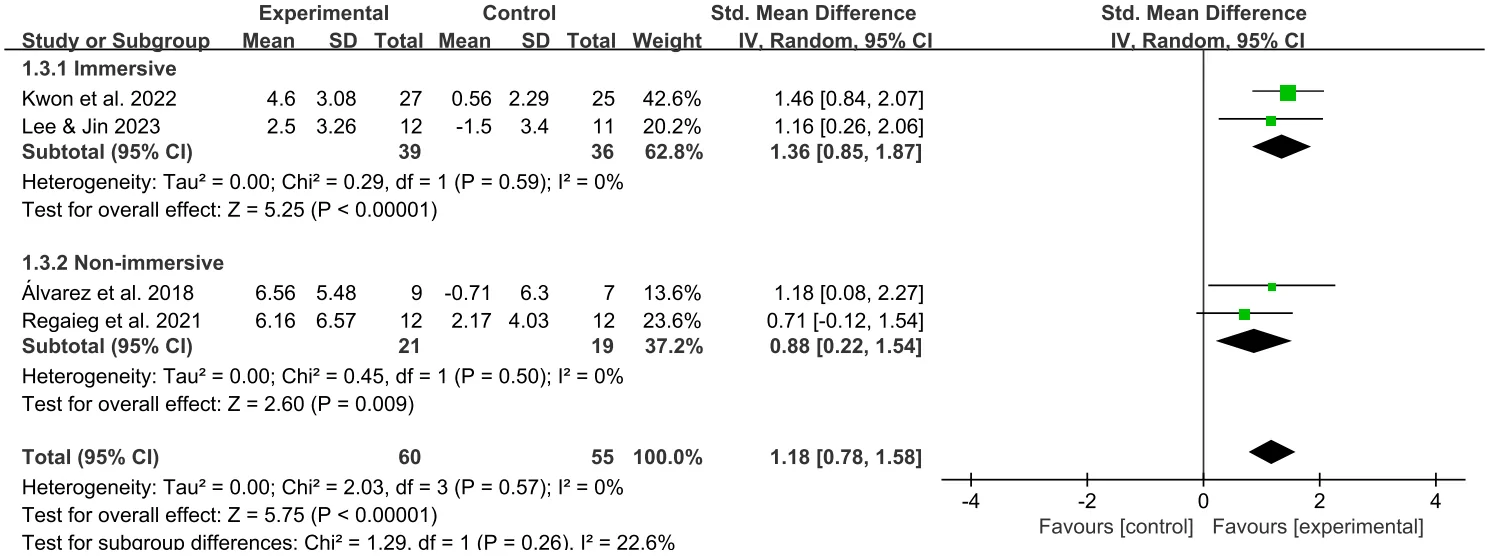
Forest plot of the effects of virtual reality on objective control skill.

The meta-analysis of object-control skill outcomes included four studies (**Figure 5**) with 60 participants in the VR groups and 55 in the control groups. The overall pooled effect indicated a statistically significant positive effect of VR interventions on object control skills (SMD = 1.18, 95% CI [0.78, 1.58], Z = 5.75, *p* < 0.00001), with no observed heterogeneity across studies (I² = 0%, *p* = 0.57). In the immersive VR subgroup, two studies involving 39 intervention participants and 36 control participants showed a statistically significant positive effect (SMD = 1.36, 95% CI [0.85, 1.87], Z = 5.25, *p* < 0.00001), with no heterogeneity (I² = 0%, *p* = 0.59). Similarly, the non-immersive VR subgroup included two studies with 21 intervention participants and 19 control participants. It also demonstrated a statistically significant positive effect (SMD = 0.88, 95% CI [0.22, 1.54], Z = 2.60, *p* = 0.009), with no observed heterogeneity (I² = 0%, *p* = 0.57). The test for subgroup differences was not statistically significant (χ² = 1, 29, *df* = 1, *p* = 0.26), indicating no significant difference between immersive and non-immersive VR interventions.

**Figure 6:**
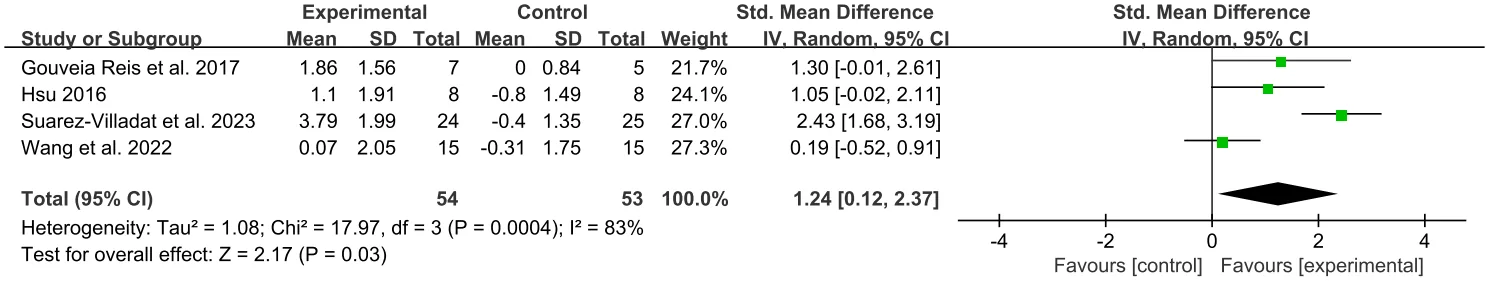
Forest plot of the effects of virtual reality on balance outcomes.

The meta-analysis comparing VR interventions with control conditions on balance outcome included four studies, comprising 54 participants in the VR groups and 53 in the control groups (**Figure 6**). The pooled effect size was statistically significant and favoured VR interventions (SMD = 1.24, 95% CI [0.12, 2.37], Z = 2.17, *p* = 0.03), suggesting a substantial positive effect on balance outcomes. Substantial heterogeneity was observed across the included studies (I² = 83%).

**Figure 7:**
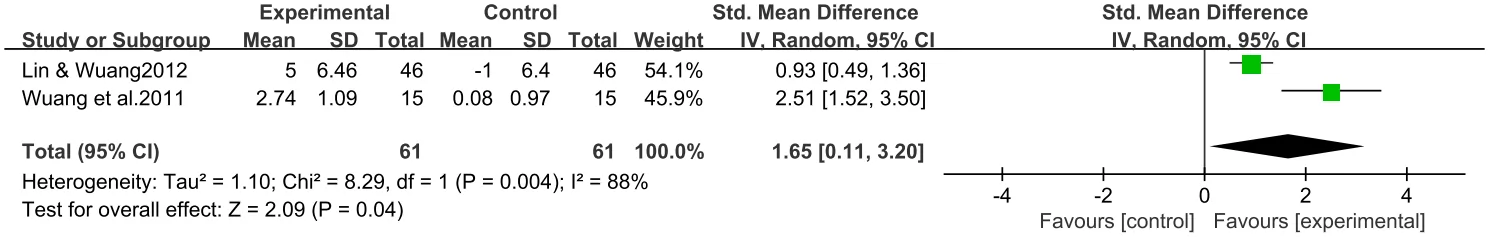
Forest plot of the effects of virtual reality on agility.

The meta-analysis comparing VR interventions with control conditions on agility included two studies, comprising 61 participants in the VR groups and 61 participants in the control groups (**Figure 7**). The pooled effect size was statistically significant and favoured VR interventions (SMD = 1.65, 95% CI [0.11, 3.20], Z = 2.09, *p* = 0.04), suggesting a substantial positive effect on agility outcomes. Substantial heterogeneity was observed across the included studies (I² = 88%).

**Figure 8:**
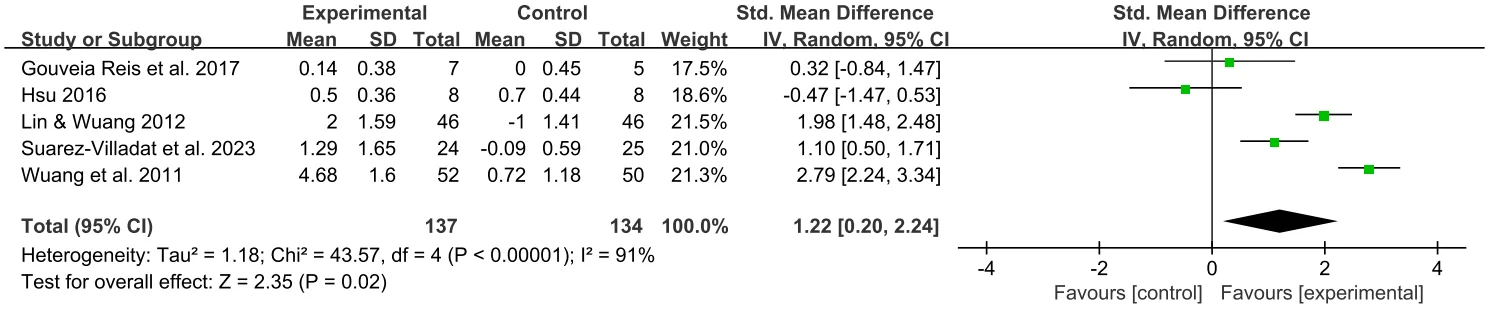
Forest plot of the effects of virtual reality on strength outcomes.

The meta-analysis comparing VR interventions with control conditions on strength outcome included five studies, comprising 137 participants in the VR groups and 134 in the control groups (**Figure 8**). The pooled effect size favoured VR interventions and indicated a positive effect on strength outcomes; the effect was statistically significant (SMD = 1.22, 95% CI [0.20, 2.24], Z = 2.35, *p* = 0.02). Substantial heterogeneity was observed across the included studies (I² = 91%).

### 3.5 Sensitivity analyses

Leave-one-out sensitivity analyses indicated that the robustness of the pooled effects varied across outcomes (**Supplementary Table 2**). For gross motor skills, all pooled estimates remained positive and statistically significant after individual studies were excluded (SMD range: 0.81–1.59), supporting the robustness of the overall finding. Excluding Lee and Jin (26) reduced heterogeneity from 69% to 0%, suggesting that this study was a potential source of heterogeneity. For locomotor skills, all estimates remained positive; however, the effect became statistically non-significant after Kwon et al. (30) was excluded (SMD = 1.32, 95% CI [−0.10, 2.75], *p* = 0.07). Moreover, excluding Lee and Jin (26) reduced heterogeneity from 74% to 0%, indicating that the locomotor finding was directionally consistent but sensitive to individual studies. The object-control finding was particularly stable, with pooled SMDs ranging from 0.98 to 1.33 and negligible heterogeneity across all analyses (I² = 0%–1%). The balance finding was also sensitive, as the pooled effect became non-significant or borderline after excluding Gouveia Reis et al. (37), Hsu (38), or Suarez-Villadat et al. (32). Excluding Suarez-Villadat et al. (32) reduced heterogeneity from 83% to 33%, identifying this study as a potential source of heterogeneity. For agility, only two studies were available; therefore, excluding either study left a single effect estimate, precluding a meaningful assessment of robustness. Finally, the strength finding was not robust, as the pooled effect became non-significant after the exclusion of Lin and Wuang (31), Suarez-Villadat et al. (32), or Wuang et al. (40). Heterogeneity remained considerable in all strength sensitivity analyses (I² = 87%–93%), suggesting that no single study fully explained the observed variability.

### 3.6 Publication bias

Formal assessment of publication bias using funnel plots or Egger’s regression test was not conducted because fewer than ten studies were included in each meta-analysis. Under such circumstances, tests for funnel plot asymmetry are considered underpowered and may yield unreliable conclusions (41).

## 4 Discussion

This systematic review and meta-analysis examined the effects of VR-based interventions on motor skills in children and adolescents with ID. Overall, the findings suggest that VR interventions have favourable effects on several motor-related outcomes, including gross motor skills, locomotor skills, object control skills, balance, agility and strength. These findings support the potential of VR as an interactive, task-oriented approach to promoting motor development in this population.

The risk-of-bias assessment showed that the overall methodological quality of the included studies was acceptable but not without limitations. Two studies were judged to be at low risk of bias (31, 32), six raised some concerns (26, 27, 36–39), and two were rated as high risk (30, 40). Randomisation procedures were generally appropriate, although one study did not clearly report whether allocation was randomised (30), and another included a non-randomly allocated no-treatment control group (40). Some concerns were also identified regarding open-label interventions, unmatched comparator intensity, and unclear assessor blinding for performance-based outcomes (26, 27, 30, 36–38). These issues may have influenced the precision and internal validity of the pooled estimates. Therefore, future studies should adopt more rigorous randomisation procedures, ensure comparable intervention exposure between groups, and use blinded outcome assessors where possible.

The positive effects of VR on gross motor skills, locomotor skills, object control skills, balance, agility and strength are consistent with previous evidence suggesting that VR and exergame-based interventions can improve motor performance in children and adolescents with ID (25, 27). These effects may be explained by several characteristics of VR-based training. First, VR environments often provide task-specific and repetitive movement practice (42). Such practice is central to motor learning because repeated exposure to goal-directed tasks helps children and adolescents refine movement patterns and improve motor coordination (43). Second, VR systems usually provide immediate visual, auditory, or performance-based feedback, which may help participants correct errors and maintain engagement during practice (44). Third, game-like tasks may increase motivation, enjoyment, and active participation, which are particularly important for children and adolescents with ID, who may experience difficulties with attention, motivation, and sustained participation in traditional exercise settings (45).

Gross motor effects remained robust, whereas the locomotor finding was less stable, losing statistical significance after the exclusion of one study. Removing Lee and Jin (26) eliminated heterogeneity for both outcomes, identifying this study as a potential source of variability. One potential contributor is its diagnostically heterogeneous sample. Lee and Jin included children with autism spectrum disorder, ID, and Down syndrome. Motor impairments in children with autism spectrum disorder are common but vary substantially in their presentation and severity (46). Moreover, autism spectrum disorder and ID are associated with heterogeneous sensory-processing profiles, which may influence participants’ engagement with and responses to multisensory VR environments (47, 48).

Object control skills showed the most consistent improvement, with no observed heterogeneity. This finding may be related to the task demands of many VR and exergame activities. Many sports-oriented VR-based or exergame tasks require reaching, throwing, catching, striking, or aiming movements. These actions are closely aligned with object control skills and may therefore produce stronger domain-specific improvements. This interpretation is consistent with the principle of training specificity, which suggests that motor improvements are more likely when training tasks closely resemble the assessed movement outcomes (49). In contrast, moderate-to-substantial heterogeneity was observed for several other outcomes. This heterogeneity may reflect differences in intervention duration and frequency, VR device type, task design, participant age, disability severity, and outcome measurement tools. Similar variability has also been noted in previous reviews of VR-based interventions in children with developmental disabilities (50).

The balance statistical significance was sensitive to the exclusion of individual studies, with Suarez-Villadat et al. (32) contributing substantially to heterogeneity. Although the favourable direction is consistent with previous reviews of children with Down syndrome and other neurodevelopmental disorders (51, 52), the larger but less stable estimate observed here may reflect the limited evidence base and differences in participant characteristics, interventions, control conditions, and outcome measures. Wang et al. (27), for example, found that an 8-week Xbox Kinect intervention improved lower-limb strength but not balance, suggesting that general movement exposure may be insufficient without adequate balance-specific practice. Overall, VR may benefit balance, but further research is needed to determine the optimal duration, frequency, intensity, and progression of postural challenges. Moreover, the positive effect on agility is consistent with the review by Li et al. (53), which reported preliminary evidence that VR-based exercise may improve speed and agility in individuals with ID. These benefits may result from the rapid movements, directional changes, and dynamic postural adjustments required during exergaming.

Although the pooled effect on strength was statistically significant, statistical significance was lost in several leave-one-out analyses, while substantial heterogeneity persisted, indicating limited robustness. This variability may partly reflect differences in training specificity and dose. Strength adaptation requires adequate mechanical loading, intensity, volume, progression, and recovery (54, 55). However, recent reviews indicate that VR interventions in this population have predominantly involved motion-controlled exergames incorporating whole-body movements, balance exercises, locomotor tasks, and simulated sports rather than structured resistance training (53, 56). Although these activities may improve neuromuscular coordination, functional performance, and muscular endurance, they may not consistently provide the progressive overload required for strength development. Differences in the definition and measurement of strength may have further contributed to the heterogeneity. Therefore, VR may benefit muscular fitness, but the magnitude and reliability of its effects on strength remain uncertain.

Effect estimates generally favoured VR in both immersive and non-immersive subgroups. Although the subgroup difference for gross motor skills favoured immersive VR, this finding was based on only one immersive study and should therefore be considered exploratory. No significant subgroup differences were observed for locomotor or object-control skills, suggesting that immersion level alone may not explain intervention effectiveness. Instead, outcomes may depend more on incorporating motor-learning principles, including task-specific practice, sufficient repetition, progressive difficulty, meaningful feedback, and sustained engagement. This interpretation is consistent with motor-learning and neurorehabilitation literature emphasising repetitive, feedback-rich, and progressively challenging practice for motor-skill acquisition and functional improvement (42, 57, 58).

From a practical perspective, this finding has important implications. Non-immersive VR may be a more accessible and feasible option for school-, home-, and community-based programmes because it is usually less expensive, easier to implement, and requires fewer safety precautions. In contrast, immersive VR may be more appropriate when a highly engaging, controlled, and multisensory virtual environment is needed. Therefore, the selection of VR format should be based not only on technological sophistication but also on intervention goals, participant characteristics, resource availability, and implementation context.

Compared with traditional physical education or adapted physical activity programmes, VR-based interventions may offer additional advantages by creating interactive, enjoyable, and multisensory learning environments. For example, Regaieg et al. (39) used Xbox 360 Kinect games with adolescents with ID and reported significant improvements in gross motor skills compared with adapted physical education. These findings suggest that VR may support motor development not only through task-specific practice but also by enhancing motivation, attention, enjoyment, and active participation.

### 4.1 Limitations

This review has several limitations. First, only ten studies, generally with small sample sizes, were included, and each outcome-specific meta-analysis contained only a small number of studies. Consequently, the pooled estimates had limited precision, the subgroup findings should be considered exploratory, and a reliable assessment of publication bias was not possible. Second, two studies enrolled diagnostically mixed samples, limiting the applicability of the findings specifically to children and adolescents with ID. Inconsistent reporting of ID severity, co-occurring conditions, baseline motor ability, and support needs further restricted the generalisability of the findings. Third, the inclusion of quasi-experimental studies, together with concerns regarding randomisation and assessor blinding, weakened causal inference. Fourth, substantial variation in VR modality, intervention dose, comparator conditions, and outcome measures limited the clinical interpretation and comparability of the pooled effects. Furthermore, sensitivity analyses demonstrated limited robustness for the locomotor, balance, and strength findings. Finally, restricting the review to English-language, peer-reviewed publications and the inability to assess funnel-plot asymmetry mean that language bias, publication bias, and small-study effects cannot be excluded.

Future research should prioritise adequately powered, preregistered randomised controlled trials using standardised outcome measures and clearly defined intervention protocols. Detailed reporting of diagnostic characteristics, impairment severity, co-occurring conditions, baseline functioning, and intervention dose would facilitate subgroup comparisons and improve clinical applicability. Future reviews should also consider non-English and grey literature and reassess publication bias as more studies become available.

## 5 Conclusion

This review provides preliminary evidence that VR-based interventions may improve motor-related outcomes in children and adolescents with ID. Favourable pooled effects were observed across gross motor, locomotor, object-control, balance, agility, and strength outcomes, although their robustness varied. Although immersive VR showed a larger effect estimate for gross motor skills than non-immersive VR, this finding was based on a single immersive study and should be considered exploratory. Given the limited evidence base, clinical heterogeneity, and methodological limitations, adequately powered and rigorously designed randomised controlled trials are needed to confirm these findings.

## Data Availability

The original contributions presented in the study are included in the article/**Supplementary Material**. Further inquiries can be directed to the corresponding author.
