# Supplementary material for: Effects of virtual reality interventions on motor skills in children and adolescents with intellectual disabilities: a systematic review and meta-analysis
Source: Front Psychiatry. 2026 Aug 25;17:1919592. doi: 10.3389/fpsyt.2026.1919592 (PMC13550863; doi:10.3389/fpsyt.2026.1919592)
Supplement: Supplementary file 1 [file SupplementaryFile1.docx]

Supplementary Material

**Supplementary Table 1.** PRISMA for Pairwise Meta-Analyses Checklist.

| **Section and Topic** | **Item #** | **Checklist item** | **Location where item is reported** |
| --- | --- | --- | --- |
| **TITLE** | | |  |
| Title | 1 | Identify the report as a systematic review. | Title, p. 1 |
| **ABSTRACT** | | |  |
| Abstract | 2 | See the PRISMA 2020 for Abstracts checklist. | Abstract, p. 1 |
| **INTRODUCTION** | | |  |
| Rationale | 3 | Describe the rationale for the review in the context of existing knowledge. | Introduction, pp. 2-3 |
| Objectives | 4 | Provide an explicit statement of the objective(s) or question(s) the review addresses. | Introduction, p. 3 |
| **METHODS** | | |  |
| Eligibility criteria | 5 | Specify the inclusion and exclusion criteria for the review and how studies were grouped for the syntheses. | Inclusion and exclusion criteria, p. 4; Data analysis, p. 5 |
| Information sources | 6 | Specify all databases, registers, websites, organisations, reference lists and other sources searched or consulted to identify studies. Specify the date when each source was last searched or consulted. | Search strategy, p. 3; Literature search results, pp. 6-7 |
| Search strategy | 7 | Present the full search strategies for all databases, registers and websites, including any filters and limits used. | Search strategy and Table 1, pp. 3-4 |
| Selection process | 8 | Specify the methods used to decide whether a study met the inclusion criteria of the review, including how many reviewers screened each record and each report retrieved, whether they worked independently, and if applicable, details of automation tools used in the process. | Study selection process, p. 5; Figure 1, p. 7 |
| Data collection process | 9 | Specify the methods used to collect data from reports, including how many reviewers collected data from each report, whether they worked independently, any processes for obtaining or confirming data from study investigators, and if applicable, details of automation tools used in the process. | Data extraction, p. 5 |
| Data items | 10a | List and define all outcomes for which data were sought. Specify whether all results that were compatible with each outcome domain in each study were sought (e.g. for all measures, time points, analyses), and if not, the methods used to decide which results to collect. | Data extraction, p. 5; Data analysis, p. 5; Meta-analysis results, pp. 10-13 |
|  | 10b | List and define all other variables for which data were sought (e.g. participant and intervention characteristics, funding sources). Describe any assumptions made about any missing or unclear information. | Data extraction, p. 5; Basic characteristics of included studies and Table 2, pp. 8-9 |
| Study risk of bias assessment | 11 | Specify the methods used to assess risk of bias in the included studies, including details of the tool(s) used, how many reviewers assessed each study and whether they worked independently, and if applicable, details of automation tools used in the process. | Risk of bias, p. 5; Quality assessment and Figure 2, p. 10 |
| Effect measures | 12 | Specify for each outcome the effect measure(s) (e.g. risk ratio, mean difference) used in the synthesis or presentation of results. | Data analysis, p. 5 |
| Synthesis methods | 13a | Describe the processes used to decide which studies were eligible for each synthesis (e.g. tabulating the study intervention characteristics and comparing against the planned groups for each synthesis (item #5)). | Inclusion and exclusion criteria, p. 4; Data analysis, p. 5; Basic characteristics of included studies and Table 2, pp. 8-9 |
|  | 13b | Describe any methods required to prepare the data for presentation or synthesis, such as handling of missing summary statistics, or data conversions. | Data extraction, p. 5; Data analysis, p. 5 |
|  | 13c | Describe any methods used to tabulate or visually display results of individual studies and syntheses. | Table 2, p. 9; Figures 3-8, pp. 11-13 |
|  | 13d | Describe any methods used to synthesize results and provide a rationale for the choice(s). If meta-analysis was performed, describe the model(s), method(s) to identify the presence and extent of statistical heterogeneity, and software package(s) used. | Data analysis, p. 5 |
|  | 13e | Describe any methods used to explore possible causes of heterogeneity among study results (e.g. subgroup analysis, meta-regression). | Data analysis, p. 5; Meta-analysis results, pp. 10-13 |
|  | 13f | Describe any sensitivity analyses conducted to assess robustness of the synthesized results. | Data analysis, p. 5; Sensitivity Analyses, pp. 13-14 |
| Reporting bias assessment | 14 | Describe any methods used to assess risk of bias due to missing results in a synthesis (arising from reporting biases). | Data analysis, p. 5; Publication bias, p. 14 |
| Certainty assessment | 15 | Describe any methods used to assess certainty (or confidence) in the body of evidence for an outcome. | NA |
| **RESULTS** | | |  |
| Study selection | 16a | Describe the results of the search and selection process, from the number of records identified in the search to the number of studies included in the review, ideally using a flow diagram. | Literature search results and Figure 1, pp. 6-7 |
|  | 16b | Cite studies that might appear to meet the inclusion criteria, but which were excluded, and explain why they were excluded. | NA |
| Study characteristics | 17 | Cite each included study and present its characteristics. | Basic characteristics of included studies and Table 2, pp. 8-9 |
| Risk of bias in studies | 18 | Present assessments of risk of bias for each included study. | Quality assessment and Figure 2, p. 10 |
| Results of individual studies | 19 | For all outcomes, present, for each study: (a) summary statistics for each group (where appropriate) and (b) an effect estimate and its precision (e.g. confidence/credible interval), ideally using structured tables or plots. | Meta-analysis results, pp. 10-13; Figures 3-8, pp. 11-13 |
| Results of syntheses | 20a | For each synthesis, briefly summarise the characteristics and risk of bias among contributing studies. | Quality assessment, p. 10; Meta-analysis results, pp. 10-13 |
|  | 20b | Present results of all statistical syntheses conducted. If meta-analysis was done, present for each the summary estimate and its precision (e.g. confidence/credible interval) and measures of statistical heterogeneity. If comparing groups, describe the direction of the effect. | Meta-analysis results, pp. 10-13; Figures 3-8, pp. 11-13 |
|  | 20c | Present results of all investigations of possible causes of heterogeneity among study results. | Meta-analysis results, pp. 10-13 |
|  | 20d | Present results of all sensitivity analyses conducted to assess the robustness of the synthesized results. | Sensitivity Analyses, pp. 13-14 |
| Reporting biases | 21 | Present assessments of risk of bias due to missing results (arising from reporting biases) for each synthesis assessed. | Publication bias, p. 14 |
| Certainty of evidence | 22 | Present assessments of certainty (or confidence) in the body of evidence for each outcome assessed. | NA |
| **DISCUSSION** | | |  |
| Discussion | 23a | Provide a general interpretation of the results in the context of other evidence. | Discussion, pp. 14-16 |
|  | 23b | Discuss any limitations of the evidence included in the review. | Discussion, pp. 14-16; Limitations, pp. 16-17 |
|  | 23c | Discuss any limitations of the review processes used. | Limitations, pp. 16-17 |
|  | 23d | Discuss implications of the results for practice, policy, and future research. | Discussion, pp. 14-16; Limitations and Conclusion, pp. 16-17 |
| **OTHER INFORMATION** | | |  |
| Registration and protocol | 24a | Provide registration information for the review, including register name and registration number, or state that the review was not registered. | Materials and methods, p. 3 |
|  | 24b | Indicate where the review protocol can be accessed, or state that a protocol was not prepared. | NA |
|  | 24c | Describe and explain any amendments to information provided at registration or in the protocol. | NA |
| Support | 25 | Describe sources of financial or non-financial support for the review, and the role of the funders or sponsors in the review. | Funding, p. 17 |
| Competing interests | 26 | Declare any competing interests of review authors. | Conflict of Interest, p. 17 |
| Availability of data, code and other materials | 27 | Report which of the following are publicly available and where they can be found: template data collection forms; data extracted from included studies; data used for all analyses; analytic code; any other materials used in the review. | NA |

*From:*  Page MJ, McKenzie JE, Bossuyt PM, Boutron I, Hoffmann TC, Mulrow CD, et al. The PRISMA 2020 statement: an updated guideline for reporting systematic reviews. BMJ 2021;372:n71. doi: 10.1136/bmj.n71

For more information, visit: <http://www.prisma-statement.org/>

**Supplementary Material 2:** Search Strategies

PubMed：

("Intellectual Disability"[Mesh] OR "Developmental Disabilities"[Mesh] OR "Down Syndrome"[Mesh] OR "Intellectual Disability"[Title/Abstract] OR "Intellectual Disabilities"[Title/Abstract] OR "Intellectual Development Disorder"[Title/Abstract] OR "Development Disorder"[Title/Abstract] OR "Intellectual Disorder"[Title/Abstract] OR "Intellectual Development Disorders"[Title/Abstract] OR "Mental Retardation"[Title/Abstract] OR "Idiocy"[Title/Abstract] OR "Psychosocial Mental Retardation"[Title/Abstract] OR "Mental Deficiencies"[Title/Abstract] OR "Mental Deficiency"[Title/Abstract] OR "mental handicap"[Title/Abstract] OR "Cognitive Disability"[Title/Abstract] OR "Cognitive Impairment"[Title/Abstract] OR "Cognitive Deficit"[Title/Abstract] OR "Cognitive Disorder"[Title/Abstract] OR "Global Developmental Delay"[Title/Abstract] OR "Developmental Delay"[Title/Abstract] OR "Cri-du-Chat Syndrome"[Title/Abstract] OR "De Lange Syndrome"[Title/Abstract] OR "Down Syndrome"[Title/Abstract] OR "Prader-Willi Syndrome"[Title/Abstract] OR "Rubinstein-Taybi Syndrome"[Title/Abstract] OR "Trisomy 13 Syndrome"[Title/Abstract] OR "WAGR Syndrome"[Title/Abstract] OR "Williams Syndrome"[Title/Abstract] OR "X-Linked Intellectual Disability"[Title/Abstract] OR "Adrenoleukodystrophy"[Title/Abstract] OR "Coffin-Lowry Syndrome"[Title/Abstract] OR "Fragile X Syndrome"[Title/Abstract] OR "Glycogen Storage Disease Type IIb"[Title/Abstract] OR "Lesch-Nyhan Syndrome"[Title/Abstract] OR "Menkes Kinky Hair Syndrome"[Title/Abstract] OR "Mucopolysaccharidosis II"[Title/Abstract] OR "Pyruvate Dehydrogenase Complex Deficiency Disease"[Title/Abstract] OR "Rett Syndrome"[Title/Abstract])

AND

("Virtual Reality"[Mesh] OR "Video Games"[Mesh] OR "Virtual Reality"[Title/Abstract] OR "Educational Virtual Realities"[Title/Abstract] OR "Educational Virtual Reality"[Title/Abstract] OR "Instructional Virtual Realities"[Title/Abstract] OR "Instructional Virtual Reality"[Title/Abstract] OR immersive[Title/Abstract] OR non-immersive[Title/Abstract] OR "Virtual Game"[Title/Abstract] OR Exergame[Title/Abstract] OR "Active Game"[Title/Abstract] OR "Active Video Game"[Title/Abstract] OR "Interactive Video Game"[Title/Abstract] OR Wii[Title/Abstract] OR Xbox[Title/Abstract] OR Kinect[Title/Abstract] OR "Play Station"[Title/Abstract] OR PlayStation[Title/Abstract] OR "Android Virtual Device"[Title/Abstract] OR Nintendo[Title/Abstract] OR Switch[Title/Abstract])

AND

("Motor Skills"[Mesh] OR "Psychomotor Performance"[Mesh] OR "Postural Balance"[Mesh] OR "Motor Skill"[Title/Abstract] OR "Motor Skills"[Title/Abstract] OR "Gross Motor Skill"[Title/Abstract] OR "Gross Motor Skills"[Title/Abstract] OR "Fine Motor Skill"[Title/Abstract] OR "Fine Motor Skills"[Title/Abstract] OR "Object Control"[Title/Abstract] OR Coordination[Title/Abstract] OR Locomotor[Title/Abstract] OR Balance[Title/Abstract] OR Agility[Title/Abstract] OR "Motor Proficiency"[Title/Abstract] OR "Motor Competency"[Title/Abstract] OR "Motor Ability"[Title/Abstract] OR "Motor Development"[Title/Abstract] OR "Movement Skill"[Title/Abstract] OR "Movement Skills"[Title/Abstract] OR "Motor Performance"[Title/Abstract] OR "Motor Fitness"[Title/Abstract] OR "Fundamental Movement"[Title/Abstract] OR "Basic Movement"[Title/Abstract] OR "Motor Coordination"[Title/Abstract] OR "Motor Control"[Title/Abstract] OR "Manual Dexterity"[Title/Abstract] OR "Motor Learning"[Title/Abstract])

AND humans [MeSH Terms]

AND English [Language]

WOS：

TS=("Intellectual Disability" OR "Intellectual Disabilities" OR “Intellectual Development Disorder" OR "Development Disorder" OR "Intellectual Disorder" OR "Intellectual Development Disorders" OR "Mental Retardation" OR "Idiocy" OR "Psychosocial Mental Retardation" OR "Mental Deficiencies" OR "Mental Deficiency" OR "mental handicap" OR "Cognitive Disability" OR "Cognitive Impairment" OR "Cognitive Deficit" OR "Cognitive Disorder" OR "Global Developmental Delay" OR "Developmental Delay" OR "Cri-du-Chat Syndrome" OR "De Lange Syndrome" OR "Down Syndrome" OR "Prader-Willi Syndrome" OR "Rubinstein-Taybi Syndrome" OR "Trisomy 13 Syndrome" OR "WAGR Syndrome" OR "Williams Syndrome" OR "X-Linked Intellectual Disability" OR "Adrenoleukodystrophy" OR "Coffin-Lowry Syndrome" OR "Fragile X Syndrome" OR "Glycogen Storage Disease Type IIb" OR "Lesch-Nyhan Syndrome" OR "Menkes Kinky Hair Syndrome" OR "Mucopolysaccharidosis II" OR "Pyruvate Dehydrogenase Complex Deficiency Disease" OR "Rett Syndrome")

AND

TS=("Virtual Reality" OR "Educational Virtual Realities" OR "Educational Virtual Reality" OR "Instructional Virtual Realities" OR "Instructional Virtual Reality" OR immersive OR non-immersive OR "Virtual Game" OR Exergame OR "Active Game" OR "Active Video Game" OR "Interactive Video Game" OR Wii OR Xbox OR Kinect OR "Play Station" OR PlayStation OR "Android Virtual Device" OR Nintendo OR Switch)

AND

TS=("Motor Skill" OR "Motor Skills" OR "Gross Motor Skill" OR "Gross Motor Skills" OR "Fine Motor Skill" OR "Fine Motor Skills" OR "Object Control" OR Coordination OR Locomotor OR Balance OR Agility OR "Motor Proficiency" OR "Motor Competency" OR "Motor Ability" OR "Motor Development" OR "Movement Skill" OR "Movement Skills" OR "Motor Performance" OR "Motor Fitness" OR "Fundamental Movement" OR "Basic Movement" OR "Motor Coordination" OR "Motor Control" OR "Manual Dexterity" OR "Motor Learning"

**Supplementary Table 2:** Leave-one-out sensitivity analysis

| **Outcome** | **Excluded study** | **Pooled SMD** | **95% CI** | ***p* value** | **I²** |
| --- | --- | --- | --- | --- | --- |
| **Gross motor skill** | **Total** | **1.28** | **0.48, 2.08** | **0.002** | **69%** |
|  | Lee & Jin, 2023 | 0.81 | 0.37, 1.25 | 0.0003 | 0% |
|  | Álvarez et al., 2018 | 1.32 | 0.26, 2.38 | 0.01 | 79% |
|  | Regaieg et al., 2021 | 1.42 | 0.35, 2.60 | 0.02 | 79% |
|  | Suarez-Villadat et al., 2023 | 1.59 | 0.65, 2.53 | 0.0009 | 59% |
| **Locomotor skill** | **Total** | **1.19** | **0.32, 2.06** | **0.007** | **74%** |
|  | Kwon et al., 2022 | 1.32 | -0.10, 2.75 | 0.07 | 83% |
|  | Lee & Jin, 2023 | 0.81 | 0.38, 1.24 | 0.0002 | 0% |
|  | Álvarez et al., 2018 | 1.35 | 0.35, 2.55 | 0.01 | 80% |
|  | Regaieg et al., 2021 | 1.40 | 0.15, 2.65 | 0.03 | 82% |
| **Object control skill** | **Total** | **1.18** | **0.78, 1.58** | **<0.00001** | **0%** |
|  | Kwon et al., 2022 | 0.98 | 0.44, 1.51 | 0.0003 | 0% |
|  | Lee & Jin, 2023 | 1.19 | 0.73, 1.64 | <0.00001 | 1% |
|  | Álvarez et al., 2018 | 1.18 | 0.74, 1.62 | <0.00001 | 0% |
|  | Regaieg et al., 2021 | 1.33 | 0.87, 1.78 | <0.00001 | 0% |
| **Balance** | **Total** | **1.24** | **0.12, 2.37** | **0.03** | **83%** |
|  | Gouveia Reais et al., 2017 | 1.23 | -0.21, 2.66 | 0.09 | 89% |
|  | Hsu, 2016 | 1.31 | -0.22, 2.83 | 0.09 | 89% |
|  | Suarez-Villadat et al., 2023 | 0.69 | -0.01, 1.40 | 0.05 | 33% |
|  | Wang et al., 2022 | 1.68 | 0.73, 2.63 | 0.0005 | 61% |
| **Agility** | **Total** | **1.65** | **0.11, 3.20** | **0.004** | **88%** |
|  | Lin & Wuang 2012 | 2.51 | 1.52, 3.50 | <0.00001 | - |
|  | Wuang et al., 2022 | 0.93 | 0.49. 1.36 | <0.00001 | **-** |
| **Strength** | **Total** | **1.22** | **0.20, 2.24** | **0.02** | **91%** |
|  | Gouveia Reis et al., 2017 | 1.41 | 0.30, 2.52 | 0.01 | 92% |
|  | Hsu 2016 | 1.63 | 0.73, 2.54 | 0.0004 | 87% |
|  | Lin & Wuang 2012 | 0.98 | -0.44, 2.41 | 0.18 | 93% |
|  | Suarez-Villadat et al., 2023 | 1.23 | -0.08, 2.53 | 0.06 | 92% |
|  | Wuang et al., 2022 | 0.81 | -0.21, 1.83 | 0.12 | 87% |
